# Telomerase activity promotes osteoblast differentiation by modulating IGF-signaling pathway

**DOI:** 10.1007/s10522-015-9596-6

**Published:** 2015-08-11

**Authors:** Hamid Saeed, Weimin Qiu, Chen Li, Allan Flyvbjerg, Basem M. Abdallah, Moustapha Kassem

**Affiliations:** Molecular Endocrinology Laboratory (KMEB), Department of Endocrinology and Metabolism, Medical Biotechnology Center, Odense University Hospital & University of Southern Denmark, SDU, 5000 Odense C, Denmark; University College of Pharmacy, Punjab University, Allama Iqbal Campus, Lahore, 54000 Pakistan; Department of Endcrinology, University Hosptial of Aarhus, 8000 Aarhus C, Denmark; Faculty of Scince, Helwan University, Cairo, Egypt; Stem Cell Unit, King Saud University, Riyadh, Saudi Arabia

**Keywords:** Telomerase, Bone marrow stromal stem cells, Osteoblasts, Bone, Aging, IGF

## Abstract

The contribution of deficient telomerase activity to age-related decline in osteoblast functions and bone formation is poorly studied. We have previously demonstrated that telomerase over-expression led to enhanced osteoblast differentiation of human bone marrow skeletal (stromal) stem cells (hMSC) in vitro and in vivo. Here, we investigated the signaling pathways underlying the regulatory functions of telomerase in osteoblastic cells. Comparative microarray analysis and Western blot analysis of telomerase-over expressing hMSC (hMSC-TERT) versus primary hMSC revealed significant up-regulation of several components of insulin-like growth factor (IGF) signaling. Specifically, a significant increase in IGF-induced AKT phosphorylation and alkaline phosphatase (ALP) activity were observed in hMSC-TERT. Enhanced ALP activity was reduced in presence of IGF1 receptor inhibitor: picropodophyllin. In addition, telomerase deficiency caused significant reduction in IGF signaling proteins in osteoblastic cells cultured from telomerase deficient mice (*Terc*^−*/*−^). The low bone mass exhibited by *Terc*^−*/*−^ mice was associated with significant reduction in serum levels of IGF1 and IGFBP3 as well as reduced skeletal mRNA expression of *Igf1, Igf2, Igf2r,Igfbp5* and *Igfbp6.* IGF1-induced osteoblast differentiation was also impaired in *Terc*^−*/*−^ MSC. In conclusion, our data demonstrate that impaired IGF/AKT signaling contributes to the observed decreased bone mass and bone formation exhibited by telomerase deficient osteoblastic cells.

**Electronic supplementary material:**

The online version of this article (doi:10.1007/s10522-015-9596-6) contains supplementary material, which is available to authorized users.

## Introduction

Osteoblast dysfunction is the main determinant of age-related decline in bone formation and bone loss (Kassem and Marie 2011). Several extrinsic factors contribute to age-related impairment in osteoblastic functions such as age-related decline in the levels of trophic hormones e.g. estrogen, growth hormone or increased oxidative stress (Almeida et al. 2007; Ernst et al. 1989; Khosla and Riggs 2005; Kveiborg et al. 2000; Manolagas 2010; Boonen et al. 1999). On the other hand, cell intrinsic deficiency in telomerase activity leading to telomere shortening and cellular senescence contributes to organismal aging (Ye et al. 2014). There is increasing evidence that telomerase deficiency and telomere shortening contribute to the in vivo age-related impairment in bone formation. We have previously demonstrated that the telomerase deficient mice (*Terc*^−*/*−^) exhibited impaired in vivo osteoblastic bone formation and reduction in bone mass (Saeed et al. 2011) caused by cell autonomous impairment of osteoblastic cells of *Terc*^−*/*−^ mice. On the other hand, telomerase activation either through pharmacological intervention (de Bernardes et al. 2011) or through gene therapy (de Bernardes et al. 2012) led to improvement in several age-related changes in mice including some increase in bone mass. At a cellular level, enhanced telomerase activity by over-expressing human telomerase reverse transcriptase (hTERT) gene in human bone marrow-derived stromal (skeletal) stem cells (hMSC) resulted in enhanced in vitro osteoblast differentiation and in vivo heterotopic bone formation (Abdallah et al. 2005; Simonsen et al. 2002).

Little is known about the regulatory mechanisms underlying the effects of telomerase activity and telomere length on osteoblast differentiation. Insulin-like growth factor 1 (IGF1) is one of the important bone anabolic factors. Skeletal IGF1 expression is reduced during aging and its levels are correlated with age-related reduction in bone mass (Liu et al. 2008; Yakar et al. 2002). The IGF system includes two ligands IGF1 and IGF2 interacting with IGF1 cognate receptor (IGFR1) and a number of IGF binding proteins (IGFBPs) (Conover 2008). IGF system has been demonstrated to regulate MSC proliferation (Langdahl et al. 1998) and osteoblast differentiation and functions (Xue et al. 2013). Several signaling pathways mediate the effects of IGFs on bone (Giustina et al. 2008). Among these, IGF1 induction of PI3K/AKT pathway is a principal regulator of osteoblast functions (Fujita et al. 2004; Levine et al. 2006; Raucci et al. 2008; Liu et al. 2007). PI3Ks are heterodimers consisting of two subunits, catalytic subunit p110 and regulatory unit p85. PI3Ks are activated by either receptor protein tyrosine kinase (RPTK) or G-protein coupled receptors (Fresno Vara et al. 2004). This activation results in the recruitment of several proteins with pleckstrin homology domain (PH) including PDK1 and AKT. The activation of AKT plays an important role in regulating osteoblast proliferation, differentiation and survival both in vitro and in vivo (Ghosh-Choudhury et al. 2002; Kawamura et al. 2007; Kawamura and Kawaguchi 2007; Mukherjee and Rotwein 2009). Interestingly, the effects of telomerase activity on several biological processes, such as, cell growth, cell cycle and apoptosis have been reported to be mediated via *PI3K/AKT* pathway (Xia et al. 2008; Kang et al. 1999). Also, AKT has been implicated in IGF1 driven telomerase activity in a multiple myeloma cell line (Akiyama et al. 2002).

In the present study, we examined whether IGF signaling mediates the biological effects of telomerase activity on osteoblastic cell differentiation and functions. We studied the effects of genetic gain and loss of telomerase functions in MSC on IGF/AKT expression and activation. We demonstrated that telomerase activation in MSC resulted in the up-regulation of IGF/IGFBPs system and IGF-induced AKT signaling. In addition, telomerase-deficiency-induced bone loss in mice was associated with reduced expression and serum levels of IGF and IGFBPs along with impaired IGF-induced AKT activation in MSC.

## Materials and methods

### Cell culture and reagents

Mouse bone marrow cells were isolated and cultured according to the protocol described by Peister et al. (Peister et al. 2004), with modifications (Post et al. 2008). Media was changed for every 3rd day. After 1–2 weeks, the cells were dissociated using Trypsin/EDTA for 4 min at 37 °C and plated according to the experimental setup.

Primary hMSCs were established from bone marrow aspirates obtained from iliac crest from healthy donors aged 25–30 years. Ethical approval was granted by the local Scientific Ethical Committee. Bone marrow mononuclear cells were isolated by low-density gradient centrifugation using Lymphoprep (Medinor, Copenhagen, Denmark) and hMSCs cultures were established by plastic adherence as described (Kassem et al. 1993).

Telomerase over-expressing human bone marrow stromal cell line known as hMSC-TERT has been established and characterized in our lab. The method of establishing hMSC-TERT and its characterization have been described in our previous publications (Abdallah et al. 2005; Simonsen et al. 2002).

IGF-1R Inhibitor, Picropodophyllin (PPP) was purchased from Santa Cruz Biotechnology, Inc, (Heidelberg, Germany).

### DNA microarray analysis

Both primary hMSCs and hMSC-TERT cells were cultured in triplicate at 3 × 10^4^ cells/cm^2^ in petri dishes in standard growth medium. When they were 90–100 % confluent, total cellular RNA was isolated from each of three independent cultures using an RNeasy Kit (QIAGEN, Valencia, CA, USA) according to the manufacturer’s instructions. First- and second-strand cDNA synthesis was performed from 8 µg total RNA using the SuperScript Choice System (Life-Technologies, Carlsbad, CA, USA) according to the manufacturer’s instructions. Subsequent hybridization and scanning of the Affymetrix GeneChip arrays were performed as described previously (Tsugawa et al. 2003a). The biotinylated targets were hybridized to human U133 Plus 2.0 Array Affymetrix GeneChip oligonucleotide arrays. Expression measures were generated and normalized using the RMA procedure (Irizarry et al. 2003; Shie et al. 2012), implemented in the Bioconductor package (www.bioconductor.org/). Data was analyzed using Ingenuity Pathway analysis knowledge base system (Ingenuity^®^ Systems, www.ingenuity.com). Signaling pathways known to play an important role in osteoblast commitment, proliferation, differentiation and function were identified among pathways that are differentially up or down regulated in comparison to control (minimum value = 2 fold).

### Mice breeding, genotyping and handling

*Terc* deficient mice (*Terc*^−*/*−^ Strain-004132) were purchased from Jackson laboratory (Maine, USA) and kept in a pathogen-free environment on standard chow. *Terc*^−*/*−^ mice were inter-crossed to generate 3rd generation *Terc*^−*/*−^ (*Terc*^−*/*−^-G3) mice, and were maintained in a C57BL/6J background. Wild type (WT) mice were employed as controls. Genotyping was performed according to the protocol recommended by Jackson laboratory (Saeed et al. 2011).

### Serum IGFs and IGFBPs measurements

Blood samples were collected from WT and *Terc*^−*/*−^ mice (*n* = 5) and serum was collected and frozen down at −80 °C. Serum IGF1 level was measured by RIA using a polyclonal rabbit antibody (Nichols Institute Diagnostics, San Capistrano, CA) and recombinant human IGF1 as standard (GE Healthcare, Little Chalfont, UK). Monoiodinated IGF1 (^125^I-labeled [Tyr31] IGF1) was obtained from Novo-Nordisk (Bagsvaerd, Denmark). Serum IGFBPs were measured by Western ligand blotting assay as described previously (Abdallah et al. 2007; Flyvbjerg et al. 1992).

### RNA isolation and quantitative real time PCR

RNA was isolated from long bones and cultured cells using Trizol^®^ method according to the manufacture’s protocol.

cDNA was synthesized from 2 μg of total RNA using a commercial revertAid H minus first strand cDNA synthesis kit (Fermentas, Helsingborg, Sweden), according to the manual’s instructions.

Gene expression was determined by quantitative real-time PCR (RT-PCR), performed in an iCycler IQ detection system (Bio-Rad, Herlev, Denmark), using IQ SYBR Green as a double strand DNA-specific fluorescent binding dye. RT-PCR was performed in a final volume of 20 μl, containing 3 μl cDNA (diluted 1:20), 20 pmol of each primer and 10 μl of IQ SYBR^®^ Green Supermix (Bio-Rad, Herlev, Denmark) according to the manual’s instruction. Expression level for each target gene was calculated using a comparative Ct method [(1/(2^∆Ct^) formula and data were represented as relative expression to Actin reference gene. Data were analysed using Microsoft Excel 2000 to generate relative expression values.

### Osteoblast differentiation

MSCs were plated at 20 × 10^3^ cells/cm^2^ in 24 well plates and induced to differentiate into osteoblasts in Iscove modified Dulbecco medium (IMDM; GIBCO, Cat. No. 21980) containing 12 % FBS (FBS; GIBCO), 100 U/ml penicillin (GIBCO), 100 µg/ml streptomycin (GIBCO) and 12 µM l-glutamine (GIBCO, Cat. No. 25030) supplemented with 10 nM dexamethasone (Sigma), 10 mM β—glycerol-phosphate (Sigma), 50 µg/ml Vitamin C (Sigma) and 10 ng/ml IGF-1. Media were changed every 3rd day until day 10.

### Cytochemical staining

#### Alkaline phosphatase (ALP) enzymatic staining

Cells were fixed with acetone/citrate buffer pH 4.2 (1.5:1) for 5 min at room temperature and stained with Napthol-AS-TR-phosphate solution for 1 h at room temperature. Napthol-AS-TR-phosphate solution consists of Napthol-AS-TR-phosphate (Sigma) diluted 1:5 in H_2_O and Fast Red TR (Sigma) diluted 1:1.2 in 0.1 M Tris buffer (OUH pharmacy), pH 9.0, after which both solutions were mixed 1:1. Cells were counterstained with Mayers-Hematoxylin for 5 min at room temperature.

#### Alkaline phosphatase activity assay

ALP activity was measured by using *p*-nitrophenyl phosphate (Fluka, Denmark) as substrate—normalized against the cell number as described previously (Qiu et al. 2010). Briefly, cell number was quantitated by adding CellTiter-Blue reagent (Promega) to culture medium, incubating at 37 °C for 1 h and measuring fluorescent intensity (560_EX_/590_EM_). Thereafter, cells were washed with phosphate-buffered saline and Tris-buffered saline, fixed in 3.7 % formaldehyde, 90 % ethanol for 30 s at room temperature, incubated with substrate (1 mg/ml of *p*-nitrophenyl phosphate in 50 mm NaHCO_3_, pH 9.6, and 1 mm MgCl_2_) at 37 °C for 20 min, and the absorbance was measured at 405 nm.

### Western blot analysis

Cells were treated for short term with IGF-1 recombinant protein (R&D, systems) and collected at different time points post treatment—washed in PBS before adding cell lysis buffer (10 mm Tris–HCl, pH 7.4, 150 mm sodium chloride, 1 % Nonidet P-40, 0.1 % SDS, 1 mm EDTA, 1 mm phenylmethylsulfonyl fluoride, 1 mm NaF, 1 mm Na_3_VO_4_), supplemented by protease inhibitor mixture (Roche Diagnostics, Mannheim, Germany). 30 µg of protein was size fractionated using SDS-PAGE (Invitrogen, Denmark) and electroblotted onto Immobilon-P membrane (Millipore, Denmark). Blots were blocked by 5 % milk powder in Tris-buffered saline (TBS, pH 8.0) for 1 h. Antibodies (total or phosphor) specific for AKT (Ser-473), PTEN (Ser380/Ther382/383), p85, PDK1 (Ser-2448) were obtained from Cell Signaling Technology (Leiden, Netherlands).

Primary antibody (1:1000) incubations were performed in 5 % milk powder TBST (TBS containing 0.1 % Tween 20) overnight followed by incubation with secondary antibody (1:1000) with horseradish peroxidase for 2 h conjugated. Proteins were visualized with the ECL system (Amersham bioscience, UK).

Western blot band area/intensity was measured as described previously (Tsugawa et al. 2003b). Bands area/intensity for AKT, PDK1 and alpha-tubulin were measured using* Image J* (Wayne Rasband, NIH, http://rsb.info.nih.gov/ij) software (Chapman et al. 2006).

### Statistical analysis

Statistical tests were performed using Microsoft Excel^®^ and Graphpad5 (Prism^®^). Pairwise comparisons were performed using student *t* test. An alpha value ≤0.05 was considered statistically significant.

## Results

### Telomerase activation in human MSC enhanced IGF and PI3K/AKT signaling

**Fig. 1:**
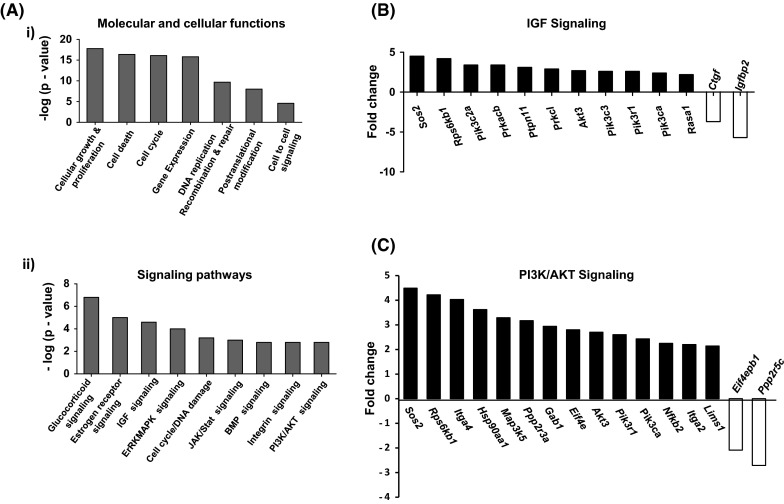
Enhanced telomerase activity in human marrow stromal cells (hMSC) Stimulates IGF/PI3K/AKT Signaling. Microarray analysis was performed for hMSC-TERT and primary isolated hMSC at baseline as described in the Materials and Methods. **a**. (*i*) Gene ontology of significantly expressed genes in hMSC-TERT in comparison to primary hMSC. (*ii*) Significantly up-regulated signaling pathways as revealed by Ingenuity^®^ pathway analysis in hMSC-TERT versus hMSC. **b** Components of IGF signaling pathway that are differentially induced by telomerase activation in hMSC. **c** Components of PI3K/AKT signaling that are differentially induced by telomerase activation in hMSC. *Black bars* represent up-regulated while *white bars* represent down-regulated pathways in hMSC-TERT versus hMSC. *p* values obtained by Fisher’s exact test and presented as—log

We have previously shown that increased telomerase activity in hMSC by stable over-expression of hTERT in hMSC-TERT cell line, resulted in enhanced cell proliferation and in vivo heterotopic bone formation (Abdallah et al. 2005; Simonsen et al. 2002). To identify signaling pathways mediating the stimulatory effect of telomeraization on hMSCs biology, we compared the molecular signature of primary hMSC where telomerase activity is absent (Simonsen et al. 2002) and hMSC-TERT cells using Affymetrix DNA micro-arrays *(unpublished data)*. Ingenuity^**®**^ pathway analysis of differentially up-regulated genes by hMSC-TERT revealed significant enrichment of components of IGF signaling (*Sos2, Pik3c2A, Akt3, Pik3c3, Igfbp4* and *Igfbp2*) and PI3 K/AKT signaling (*Akt3, Pik3r1, Pik3cA, Sos2, Itga4* and *Eif4E*) (Fig. 1a(ii), B-C, 1SB).

### Enhanced telomerase activity in hMSC increased PI3K/AKT signaling and AKT phosphorylation

**Fig. 2:**
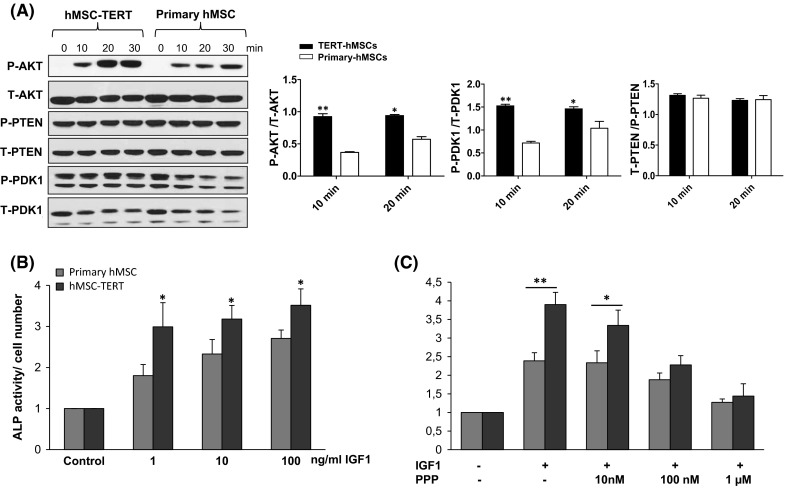
Enhanced telomerase activity in hMSC promotes IGF-induced osteoblast differentiation and activation of AKT signaling. **a** Enhanced telomerase activity in hMSC-TERT increased AKT phosphorylation following IGF1 treatment. Western blot analysis of phosphorylated and total proteins of AKT, PTEN and PDK1 following 5, 10 and 20 min of IGF1 treatment (10 ng/ml). Quantification analysis of WB was performed by ImageJ. **b** Alkaline phosphatase (ALP) activity normalized for cell number in primary hMSC and hMSC-TERT following treatment with IGF1 (dose range 1–100 ng/ml). **c** Primary hMSC and hMSC-TERT were treated with IGF1 (100 ng/ml) in the absence or the presence of IGF1R specific inhibitor (PPP). After 6 days of induction, ALP activity was measured and normalized to cell number per each treatment condition. Data are mean ± SD of three independent experiments. *p ≤ 0.05, **p ≤ 0.01

A number of studies have shown that AKT activation promotes cell proliferation, survival and differentiation in osteoblasts (Ghosh-Choudhury et al. 2002; Kawamura et al. 2007; Kawamura and Kawaguchi 2007; Mukherjee and Rotwein, 2009). To verify the activation of IGF signaling pathway by telomerase activity, we studied the activation of IGF downstream target proteins in hMSC-TERT versus primary hMSCs. As shown in Fig. 2a, Western blot analysis revealed a significant up-regulation of IGF-induced AKT phosphorylation in hMSC-TERT as compared to primary hMSCs. A significant up-regulation of p-PDK1 after 10 min of IGF1 stimulation was also observed (Fig. 2a). On the other hand, no detectable significant changes were observed in PTEN protein levels (Fig. 2a). IGF-induced osteoblast differentiation was significantly enhanced in hMSC-TERT compared to primary hMSC as assessed by increased alkaline phosphatase (ALP) activity measurements (Fig. 2b). To ensure that the effect on osteoblast differentiation was specific for IGF signaling, we examined the effect of specific IGF1R inhibitor picropodophyllin (PPP) on IGF-induced ALP activity in both primary hMSCs and hMSC-TERT cells. As shown in Fig. 2c, treatment with PPP led to a dose-dependent inhibition of IGF-induced ALP activity with more pronounced effect in hMSC-TERT than primary hMSC.

### Telomerase deficient mice exhibited changes in serum levels of IGF and its binding proteins (IGFBPs)

**Fig. 3:**
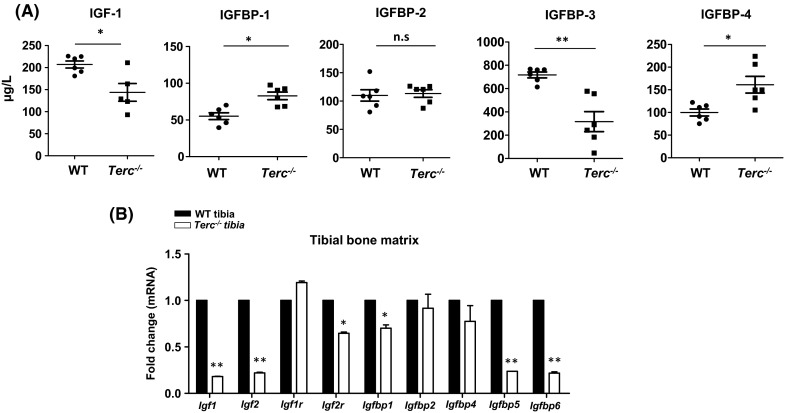
Telomerase deficiency in mice is associated with changes in levels of IGFs and IGFBPs. **a** Serum levels of IGF-1 and IGFBPs (IGFBP1-4*)* in telomerase deficient mice *Terc* ^−*/*−^ and wild type controls. **b** Gene expression of *Igfs* (*Igf*-*1*, *Igf*-*2*, *Igf*-*1r* and *Igf*-*2r*) and *Igfbps* (*(IGFBP1*-*6)* in *Terc* ^−*/*−^ tibia compared to WT controls. Each target gene was normalized to b-actin and represented as fold change over WT controls. Data are mean ± SD. *p ≤ 0.05, **p ≤ 0.01. (*n* = 5)

IGF1/IGFBP3 axis has been reported to regulate bone growth and bone mass (Schmid et al. 1991; Yakar et al. 2002). Likewise, reduced serum levels of IGF1 and IGFBP3 have been implicated in the pathogenesis of age-related osteoporosis (Sugimoto et al. 1997). To study the effect of loss of telomerase activity on IGF system, we measured serum levels of IGF1 and its binding proteins (IGFBPs) in *Terc*^−*/*−^ mice and WT controls. As shown in Fig. 3a, serum IGF1 and IGFBP3 were significantly decreased in *Terc*^−*/*−^ mice. Since production of IGFs in local tissue microenvironment plays important role in bone biology (Sjogren et al. 1999), we also measured mRNA expression of *Igf1, Igf2* and *Igfbp1*, *Igfbp5, Igfbp6* in bone matrix. We found that their expression was significantly reduced in the bone matrix of *Terc*^−*/*−^ mice (Fig. 3b).

### *Terc*^−*/*−^ mMSCs exhibits defective IGF signaling

**Fig. 4:**
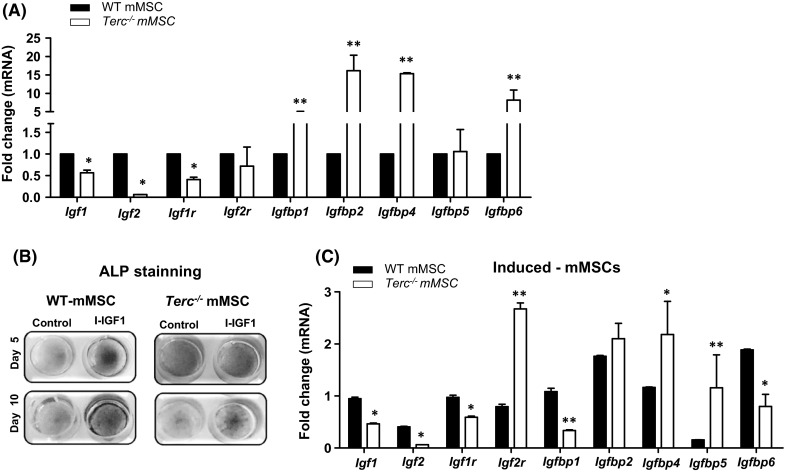
Inhibition of IGF1-induced osteoblast differentiation in telomerase deficient murine marrow stromal cells (mMSCs). **a** Real time PCR gene expression analysis of *Igf1, Igf2, Igf1r, Igf2r, Igfbp1, Igfbp2, Igfbp4, Igfbp5* and *Igfbp6* in *Terc* ^−*/*−^ mMSCs versus WT mMSCs at baseline. Each target gene was normalized to b-actin and represented as fold change over WT mMSCs. B.Alkaline phosphatase (ALP) staining at day 5 (*upper panel*) and at day 10 (*lower panel*) following IGF-1 induced osteoblast differentiation in *Terc* ^−*/*−^ mMSCs versus WT mMSCs. C. Real time PCR analysis of *Igfs, IGF receptors (IGFr)* and IGF binding proteins (*IGFBPs*) expression after 7 days of IGF-1 induction in *Terc* ^−*/*−^ mMSCs versus WT mMSC. Each target gene was normalized to b-actin and represented as fold change over control non-induced cells. Data are mean ± SD. *p ≤ 0.05, **p ≤ 0.01. (*n* = 3)

We next examined gene expression profiles of *Igf/Igfbp* system in murine MSC (mMSC) cultured from bone marrow samples obtained from *Terc*^−*/*−^ and WT control mice. At baseline, *Terc*^−*/*−^ mMSCs exhibited reduced mRNA expression of *Igf1, Igf2, Igf1receptor* (*Igf1r*) and higher expression of *Igfbp1, Igfbp2, Igfbp4* and *Igfbp6* as compared to WT mMSC (Fig. 4a). Following in vitro osteoblast differentiation in the presence of IGF1, the *Terc*^−*/*−^ MSCs exhibited impaired osteoblast differentiation in response to IGF treatment as observed by reduced staining for ALP (Fig. 4b) and reduced expression levels of *Igf1, Igf2, Igf1r, Igfbp1* and *Igfbp6*, while, expression of *Igf2r, Igfbp4* and *Igfbp5* were increased (Fig. 4c).

**Fig. 5:**
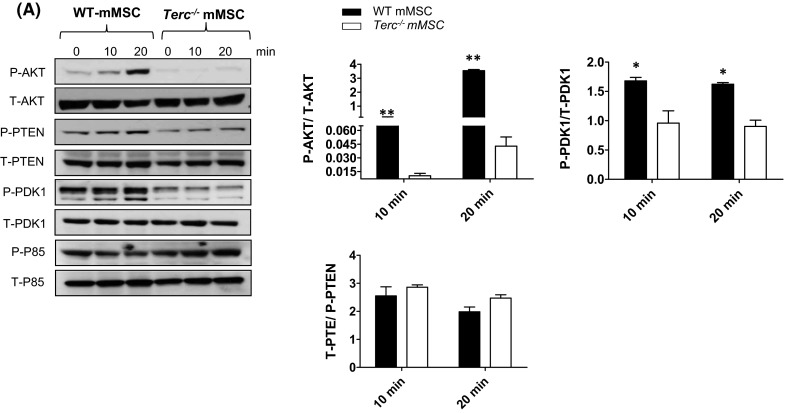
Impaired IGF1-induced AKT phosphorylation in telomerase deficient murine marrow stromal cells (mMSCs). **a** Reduced AKT and PDK1 phosphorylation following IGF1 (10 ng/mL) treatment of *Terc* ^−*/*−^ mMSCs versus WT mMSCs. Western blot analysis of phosphorylated and total p85, PTEN, PDK1 and AKT as well as loading control α-Tubulin following 10 and 20 min of IGF1stimulation. Data are generated from pooled protein samples from three independent experiments of IGF1–stimulated *Terc* ^−*/*−^ mMSCs versus WT mMSC. Quantification analysis of WB was performed by ImageJ. Data are mean ± SD of three independent experiments. *p ≤ 0.05, **p ≤ 0.01

To study the mechanism underlying the impairment of IGF-induced osteoblast differentiation in *Terc*^−*/*−^ mMSCs, we treated WT mMSCs with IGF (10 ng/ml) that resulted in increased levels of P-AKT and its downstream target P-PDK1 (Fig. 5a). In contrast IGF-induced AKT phosphorylation was absent in *Terc*^−*/*−^ mMSCs (Fig. 5a). No detectable differences in total PTEN and P-PTEN were observed between *Terc*^−*/*−^ MSCs and WT-MSCs following IGF1 stimulation (Fig. 5a).

## Discussion

In the present study, we identified IGF signaling as a downstream target of telomerase activity in skeletal stem cells (MSC). Mice lacking telomerase activity exhibited impaired osteoblastic functions and low bone mass that were associated with reduction in IGF1/IGFBP3 serum levels and impairment of IGF/AKT signaling in MSCs. Our data suggest a link between the absence of telomerase activity and the impairment of IGF signaling in MSC as a contributing factor for age-related reduced osteoblast function and bone formation.

We have previously demonstrated, in a number of gain and loss of function studies, an association between telomerase activity, telomere length and skeletal tissue homeostasis (Abdallah et al. 2005; Simonsen et al. 2002; Saeed et al. 2011). Over-expression of telomerase in hMSC not only extended their life span but also enhanced their in vivo heterotopic bone formation capacity (Simonsen et al. 2002). Telomerase deficiency in *Terc*^−*/*−^ mice resulted in age-related reduction in bone mass caused by defective osteoblast differentiation and creation of pro-inflammatory bone marrow micro-environment favoring osteoclast formation (Saeed et al. 2011). However, the target of telomerase activity in MSCs is not known.

We observed up-regulation of IGF-related genes including AKT phosphorylation in response to increased telomerase activity in hMSC suggesting the involvement of IGF/AKT signaling pathway in mediating the telomerase functional effects. The association between telomerase activity and PI3 K/AKT signaling pathway has previously been reported in a number of cellular models including growth hormone dependent telomerase activation via PI3 K signaling in Chinese hamster ovary cells (Gomez-Garcia et al. 2005). Also, transcriptional activation of hTERT in human T cell leukemia—lymphoma virus-1 infected cells was mediated by PI3K (Bellon and Nicot 2008) and PI3K/AKT-dependent activation of hTERT was reported to be accomplished by ectopic expression of EGFR in oral squamous epithelial cells (Heeg et al. 2011). Finally, increased telomerase activity in natural killer cells after IL2 stimulus took place via PI3K/AKT signaling (Kawauchi et al. 2005). Interestingly, a number of studies have shown that PI3K/AKT signaling pathway regulates hTERT activity (Breitschopf et al. 2001; Haendeler et al. 2003; Kang et al. 1999). Thus, it is plausible that telomerase activity is regulated by PI3K/AKT signaling via a feedback mechanism during bone formation that requires recruitment and proliferation of MSC at bone formation sites.

We observed that serum levels of IGF1 and IGFBP3 were reduced in telomerase deficient mice Several human studies have reported an association between decreased serum IGF1 and age-related reduction in bone mass and bone strength (Barrett-Connor and Goodman-Gruen 1998; Langlois et al. 1998; Yamaguchi et al. 2006; Garnero et al. 2000; Ohlsson et al. 2011; Rosen et al. 1997). It has also been reported that IGF1 production in bone microenvironment is more relevant to skeletal maintenance than circulating IGF1 (Ohlsson et al. 2011; Yakar et al. 2002). Our finding of the presence of low levels of gene expression of IGF system within bone matrix in telomerase deficient murine MSC corroborate the contribution of local IGF system to impaired bone formation during aging. On the other hand, mutant mice exhibiting reduced GH/IGF1/insulin secretions exhibit extended life span (Brown-Borg et al. 1996; Kurosu et al. 2005). However, bone mass has not been directly measured in these mice.

The role of IGF1 in cellular senescence is not completely understood. Similar to our findings, age-related decline in IGF1 and IGFBP3 has been reported in mice and rats (Severgnini et al. 1999; Xian et al. 2012). Also, a recent study employing aged mice reported that activation of cellular telomerase activity delayed many aspects of age-related phenotypes and improved longevity which was associated with increased serum IGF1 levels (de Bernardes et al. 2012). The role of IGF1 in bone biology has been demonstrated in IGF1-deficient mice that exhibited skeletal malformation, delayed mineralization, decreased chondrocyte proliferation and increased chondrocyte apoptosis (Laviola et al. 2008). In human conditions of bone loss, IGF1 action and signaling is impaired in osteoblastic cells (Perrini et al. 2010). Likewise, reduction in IGF1 levels has been reported in age related bone loss, both men and women (Boonen et al. 1999; Nicolas et al. 1994; Rosen et al. 1998; Rosen 2004; Seck et al. 2001). Administration of recombinant human IGF1 has been associated with improved osteoblast functions in healthy females exhibiting decreased bone turnover due to caloric restrictions (Grinspoon et al. 1996). Interestingly, PI3 K/AKT signaling is utilized by IGF1 to reduce osteoblast apoptosis (Grey et al. 2003). Comparative analysis of IGF signaling in human osteoblast cultures established from healthy donors and osteoporotic patients revealed that IGFR1 and components of IGF1 downstream signaling were not responsive to IGF1 stimulation in osteoporotic cells due to reduced phosphorylation of AKT at Ser-473 and Thr-308 (Perrini et al. 2008). Furthermore, data from IGF1 conditional gain and loss of function studies in mice employing osteoblast specific gene promoters, such as Col1a1 and Col1a2, respectively, suggest that IGF1 gain of function resulted in increase length of long bones and cortical width in mice while loss of function resulted in decrease bone formation and bone mass (Jiang et al. 2006). Similarly, Xian et al., demonstrated that IGF1 stimulation activates mTOR via PI3K/AKT pathway in Sca1^+^mMSCs and conditioned media containing IGF1 released from bone matrix significantly enhanced osteoblast differentiation and increased phosphorylation of PI3K, AKT and mTOR (Xian et al. 2012), implicating PI3K/AKT signaling in mediating IGF1 effects on bone. Thus, the ability of IGF1 to rescue aspects of age-related phenotype requires further investigation.

In addition to changes in IGF1, we observed that the levels of telomerase activity in MSC affected several IGFBPs gene expression and serum levels. The bioactivity of IGF1 is regulated by several IGF binding proteins (IGFBPs) which mainly modulate IGF1 bioavailability to IGF receptors (Muck et al. 2008). IGFBP3 is an abundant protein in human serum and binds over 90 % of circulating IGF1 resulting in prolonged half-life of IGF1 (Baxter and Martin 1989) and the resulting IGF1/IGFBP3 complex exerts tissue and cell type specific effects (Baege et al. 2004). IGFBP3 has been shown to inhibit cell growth by interfering with IGF1 interaction with its cognate receptor (Kelley et al. 1996). IGFBP3 has also IGF1-indpenent effects on cell growth (Firth and Baxter 2002). Increased expression of IGFBP3 has been reported in senescent cells (Baege et al. 2004; Muck et al. 2008) and has been proposed as a marker of cellular senescence (Baege et al. 2004). Additionally, we also observed increased in serum levels of IGFBP4 which is an inhibitory IGFBP (Kassem et al. 1996). Thus, it is possible that the net effects are decreased serum IGF1 bioactivity.

## Conclusion

Our data suggest that telomerase activity regulates IGF1 dependent PI3K/AKT signaling and that age-related deficiency in telomerase activity in osteoblastic cells contributes to age-related decreased bone formation. Modulating osteoblastic cells telomerase enzyme or IGF signaling activity pharmacologically is a possible strategy for prevention of age related bone loss.

## Electronic supplementary material

Supplementary material 1 (PDF 221 kb). Fig. S1. Differential gene expression in hMSC-TERT compared to primary hMSC. Microarray analysis was performed for hMSC-TERT and primary isolated hMSC at baseline. **a** Pie chart showing number of genes that are differentially regulated in hMSC-TERT compared to hMSCs. **b** Components of IGF signaling pathway that exhibited significant changes in hMSC-TERT. Up-regulated genes are represented in *red color*, while down-regulated genes are represented in *green color*

Supplementary material 2 (PDF 12 kb). Genes are clustered according to biological function and involvement in relevant signaling Pathways
