# Supplementary figures and images for: Telomerase activity promotes osteoblast differentiation by modulating IGF-signaling pathway
Source: Biogerontology. 2015 Aug 11;16(6):733–45. doi: 10.1007/s10522-015-9596-6 (PMC4602053; doi:10.1007/s10522-015-9596-6)

**A**

| Response     | Count | Percentage |
|--------------|-------|------------|
| More than 10 | 2145  | 82%        |
| 10 or fewer  | 465   | 18%        |

■ Down-regulated

## IGF Signaling

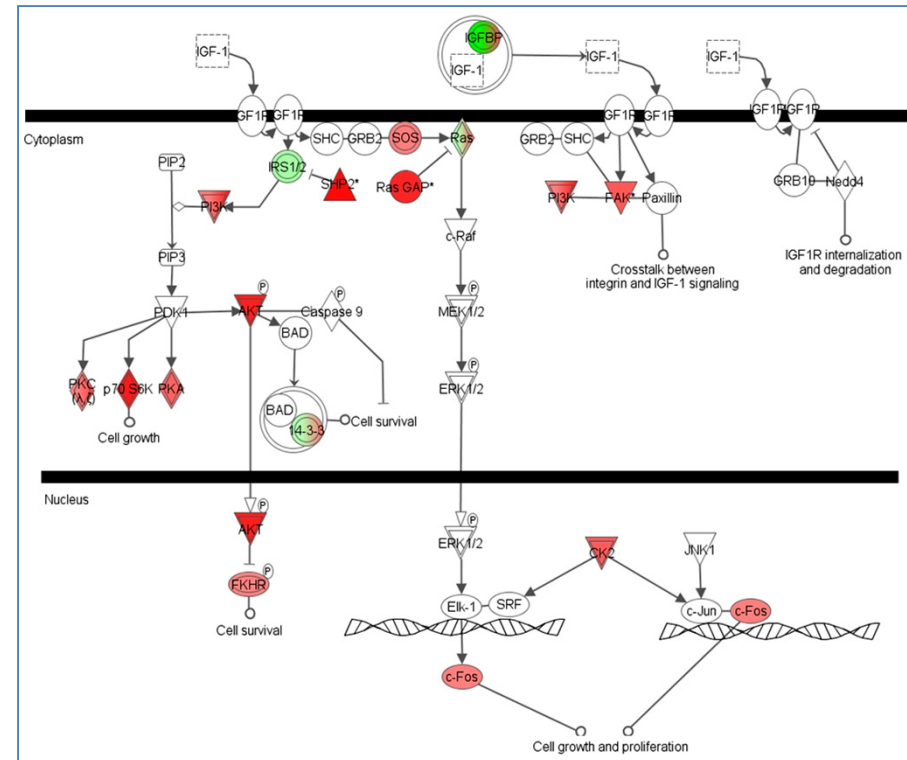

Supplement: Supplementary file 1 — Supplementary material 1 (PDF 221 kb). Fig. S1. Differential gene expression in hMSC-TERT compared to primary hMSC. Microarray analysis was performed for hMSC-TERT and primary isolated hMSC at baseline. a Pie chart showing number of genes that are differentially regulated in hMSC-TERT compared to hMSCs. b Components of IGF signaling pathway that exhibited significant changes in hMSC-TERT. Up-regulated genes are represented in red color, while down-regulated genes are represented in green color [file 10522_2015_9596_MOESM1_ESM.pdf]
