# Supplementary material for: Telomerase activity promotes osteoblast differentiation by modulating IGF-signaling pathway
Source: Biogerontology. 2015 Aug 11;16(6):733–45. doi: 10.1007/s10522-015-9596-6 (PMC4602053; doi:10.1007/s10522-015-9596-6)
Supplement: Supplementary file 2 — Supplementary material 2 (PDF 12 kb). Genes are clustered according to biological function and involvement in relevant signaling Pathways [file 10522_2015_9596_MOESM2_ESM.pdf]

Table. S1. List of Differentially Expressed Genes IGF signalling pathway related genes by Telomerase Over-expression in hMSC

IGF Signaling

Accession Number	*Gene Symbol*	*Gene Name*	*Fold Change*
**NM_006939.2**	**SOS2**	Son of sevenless homolog 2	4,49
**NM_003161.3**	**RPS6KB1**	Ribosomal protein S6, kinase, 70kDa, polypeptide 1	4,22
**NM_002645.2**	**PIK3C2A**	Phosphatidyl inositol-4-phosphate 3-kinase, catalytic subunit type 2 alpha	4,02
**NM_002731.2**	**PRKACB**	Protein kinase, cAMP dependent, catalytic, beta	3,37
**NM_002834.3**	**PTPN11**	Protein tyrosine phosphatase, non-receptor type 11	3,34
**NM_006154.3**	**NEDD4**	Neural precursor cell expressed, developmentally down regulated 4, E3 ubiquitin protein ligase	3,12
**NM_002740.5**	**PRKCI**	Protein kinase C, iota	2,93
**NM_005465.4**	**AKT3**	v-akt murine thyoma viral oncogene homolog 3	2,7
**NM_181523.2**	**PIK3R1**	Phosphoinositide-3-kinase, regulatory subunit 1	2,6
**NM_006218.2**	**PIK3CA**	Phosphatidyl inositol-4, 5-bi-phosphate 3-kinase, catalytic subunit alpha	2,43
**NM_002890.2**	**RASA1**	RAS p21 protein activator (GTPase activating protein) 1	2,21
**NM_000596.2**	**IGFBP1**	Insulin-like growth factor binding protein 1	1,79
**NM_000597.2**	**IGFBP2**	Insulin like growth factor binding protein 2, 36kDa	-5,79
**NM_001901.2**	**CTGF**	Connective tissue growth factor	-3,75
**NM_003749.2**	**IRS2**	Insulin receptor substrate 2	-1,9
**NM_001552.2**	**IGFBP4**	Insulin like growth factor binding protein 4	-1,81
**NM_003405.3**	**YWHAH**	Tyrosin 3-monooxygenase/tryptophan 5-monooxygenase activation protein. eta	-1,65
**NM_000599.3**	**IGFBP5**	Insulin like growth factor binding protein 5	-1,65

PI3K/AKT Signaling

Accession Number	*Gene Symbol*	*Gene Name*	*Fold Change*
**NM_006939.2**	**SOS2**	Son of sevenless homolog 2	4,49
**NM_003161.3**	**RPS6KB1**	Ribosomal protein S6, kinase, 70kDa, polypeptide 1	4,22
**NM_000885.4**	**ITGA4**	Integrin alpha 4 (antigen CD49D)	4,03
**NM_005348.3**	**HSP90AA1**	Heat shock protein 90kDa alpha (cytosolic), class A, member 1	3,62
**NM_005923.3**	**MAP3K5**	Mitogen activated protein kinase kinase 5	3,29
**NM_002718.4**	**PPP2R3A (includes EG:5523)**	Protein phosphatase 2, regulatory subunit B, alpha	3,17
**NM_002039.3**	**GAB1**	GAB 2 associated binding protein 1	2,94
**NM_001968.3**	**EIF4E**	Eukaryotic translation initiation factor 4E	2,8
**NM_005465.4**	**AKT3**	v-akt murine thyoma viral oncogene homolog 3	2,7
**NM_181523.2**	**PIK3R1**	Phosphoinositide-3-kinase, regulatory subunit 1	2,6

**NM_006218.2**	**PIK3CA**	Phosphatidyl inositol-4, 5-bi-phosphate 3-kinase, catalytic subunit alpha	2,43
**NM_002502.5**	**NFKB2**	Nuclear factor of kappa light polypeptide gene enhancer in B cells 2 (p49/p100)	2,25
**NM_002203.3**	**ITGA2**	Interin alpha 2 (CD49B, alpha 2 subunit of VLA-2 receptor)	2,2
**NM_004987.5**	**LIMS1**	LIM and senescent cell antigen –like domains 1	2,14
**NM_004095.3**	**EIF4EBP1**	Eukaryotic translation initiation factor 4E binding protein 1	-2,09
**NM_002719.3**	**PPP2R5C**	Protein phosphatase 2, regulatory subunit B, gamma	-2,71

PTEN Signaling

Accession Number	***Gene Symbol***	***Gene Name***	***Fold Change***
NM_006939.2	SOS2	Son of sevenless homolog 2	4,49
NM_003161.3	RPS6KB1	Ribosomal protein S6 kinase, 70kDa, polypeptide 1	4,22
NM_000885.4	ITGA4	Integrin alpha 4 (antigen CD49D)	4,03
NM_005228.3	EGFR	Epidermal growth factor receptor	4,02
NM_017742.4	ZCCHC2	Zinc finger CCHC domain containing 2	2,9
NM_006206.4	PDGFRA	Platelet derived growth factor receptor alpha polypeptide	2,89
NM_005465.4	AKT3	v-akt murine thyoma viral oncogene homolog 3	2,7
NM_181523.2	PIK3R1	Phosphoinositide-3-kinase, regulatory subunit 1	2,6
NM_006218.2	PIK3CA	Phosphatidyl inositol-4, 5-bi-phosphate 3-kinase, catalytic subunit alpha	2,43
NM_002502.5	NFKB2	Nuclear factor of kappa light polypeptide gene enhancer in B cells 2 (p49/p100)	2,25
NM_002203.3	ITGA2	Interin alpha 2 (CD49B, alpha 2 subunit of VLA-2 receptor)	2,2
NM_005249.4	FOXG1	Fork head box G1	1,98
NM_004346.3	CASP3	Caspase 3, apoptosis related cystein peptidase	1,93
NM_001278.3	CHUK	Concerve helix loop helix ubiquitious kinase	1,91
NM_000314.4	PTEN	Phosphatase and tensin homolog	1,74
NM_000163.4	GHR	Growth hormone receptor	-2,88
NM_002609.3	PDGFRB	Platelet derive growth factor receptor, beta polypeptide	-2,14
NM_003405.3	YWHAH	Tyrosin 3-monooxygenase/tryptophan 5-monooxygenase activation protein. eta	-1,65
